# Supplementary material for: Hippocampal and orbitofrontal neurons contribute to complementary aspects of associative structure
Source: Nat Commun. 2024 Jun 20;15:5283. doi: 10.1038/s41467-024-49652-9 (PMC11190210; doi:10.1038/s41467-024-49652-9)
Supplement: Supplementary file 1 — Supplementary Information [file 41467_2024_49652_MOESM1_ESM.pdf]

Hippocampal and orbitofrontal neurons contribute to complementary aspects of associative structure

Huixin Lin[1, 2], Jingfeng Zhou[2 *]

[1]Academy for Advanced Interdisciplinary Studies, Peking University, Beijing 100871, China

[2]Chinese Institute for Brain Research, Beijing 102206, China

[*]e-mail: jingfeng.zhou@cibr.ac.cn

Supplementary information in this PDF file contains Supplementary Figures 1–18 with legends and Supplementary Table 1.

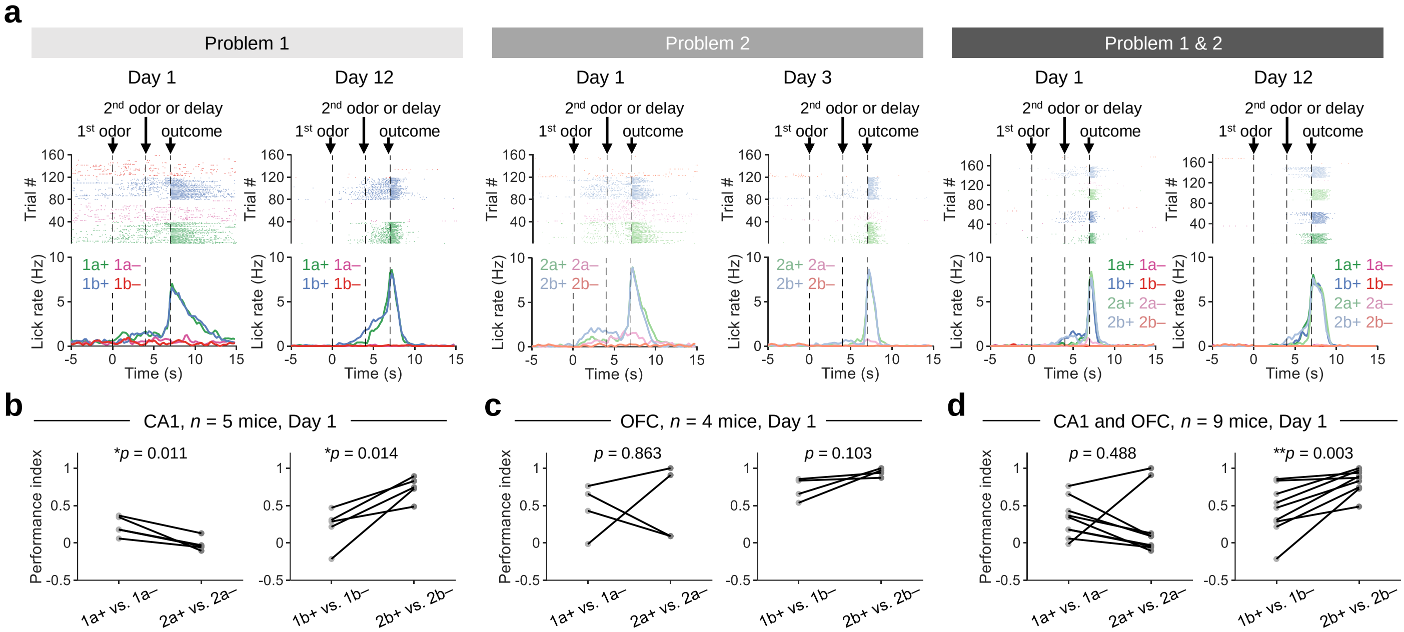

Supplementary Fig. 1 | Behavioral discrimination of paired sequences with or without a common odor. **a** Behavioral examples from one mouse throughout training. Raters represent licks. **b**-**d** Comparisons of behavioral performance index between sequence pairs on Day 1 when these sequence pairs were first introduced in the CA1 group (**b**), OFC group (**c**), and two groups combined (**d**). Each dot represents data from one single mouse. Two mice were left out for their lack of Problem 2 training in the CA1 group. Paired t-test was used to test statistical differences (*$p < 0.05$, **$p < 0.01$).

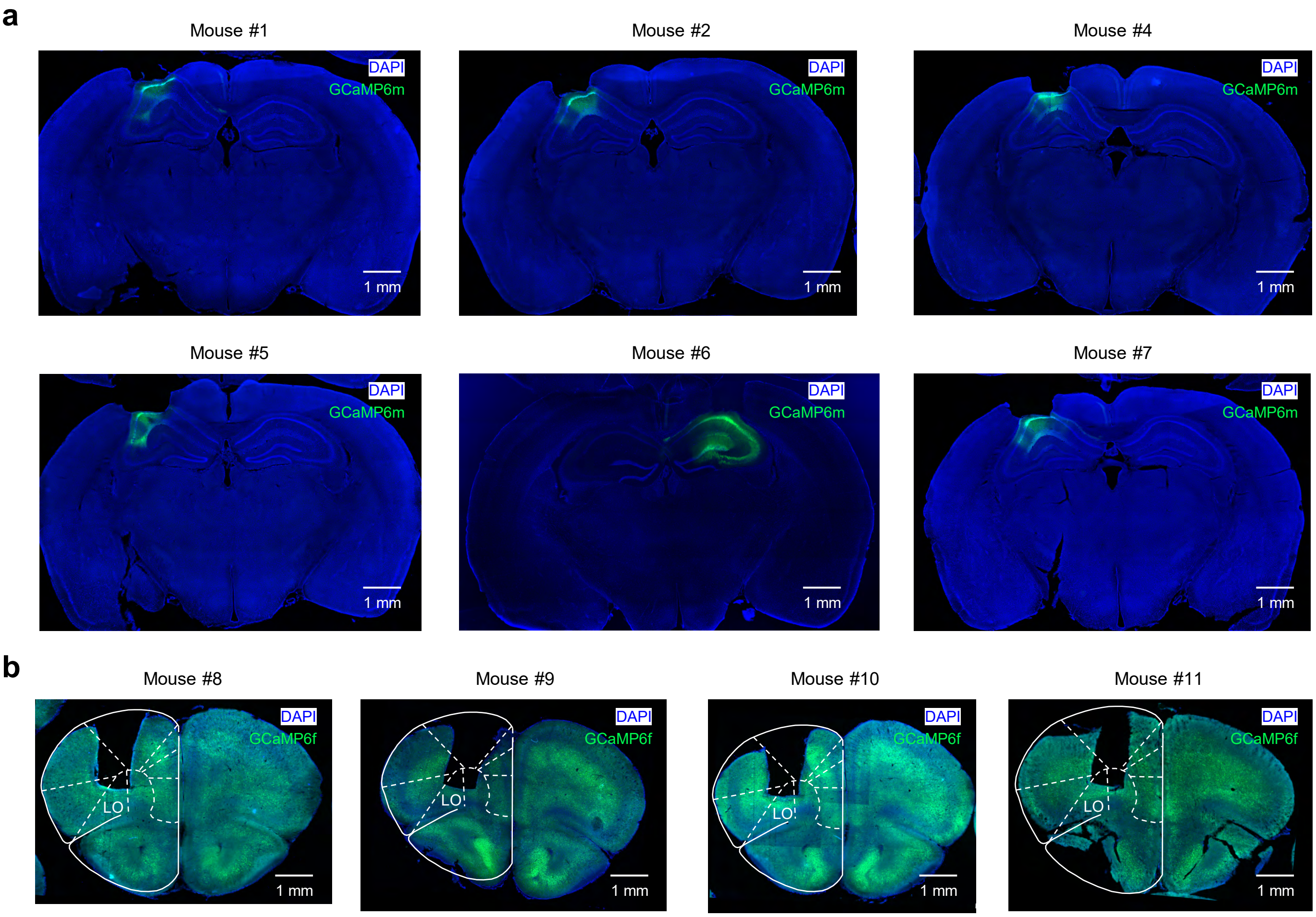

Supplementary Fig. 2 | Histology. a Virus expression (AAV-hSyn-GCaMP6m) and GRIN lens placement in the dorsal CA1. **b** GRIN lens placement in the OFC of Thy1-GCaMP6f mice.

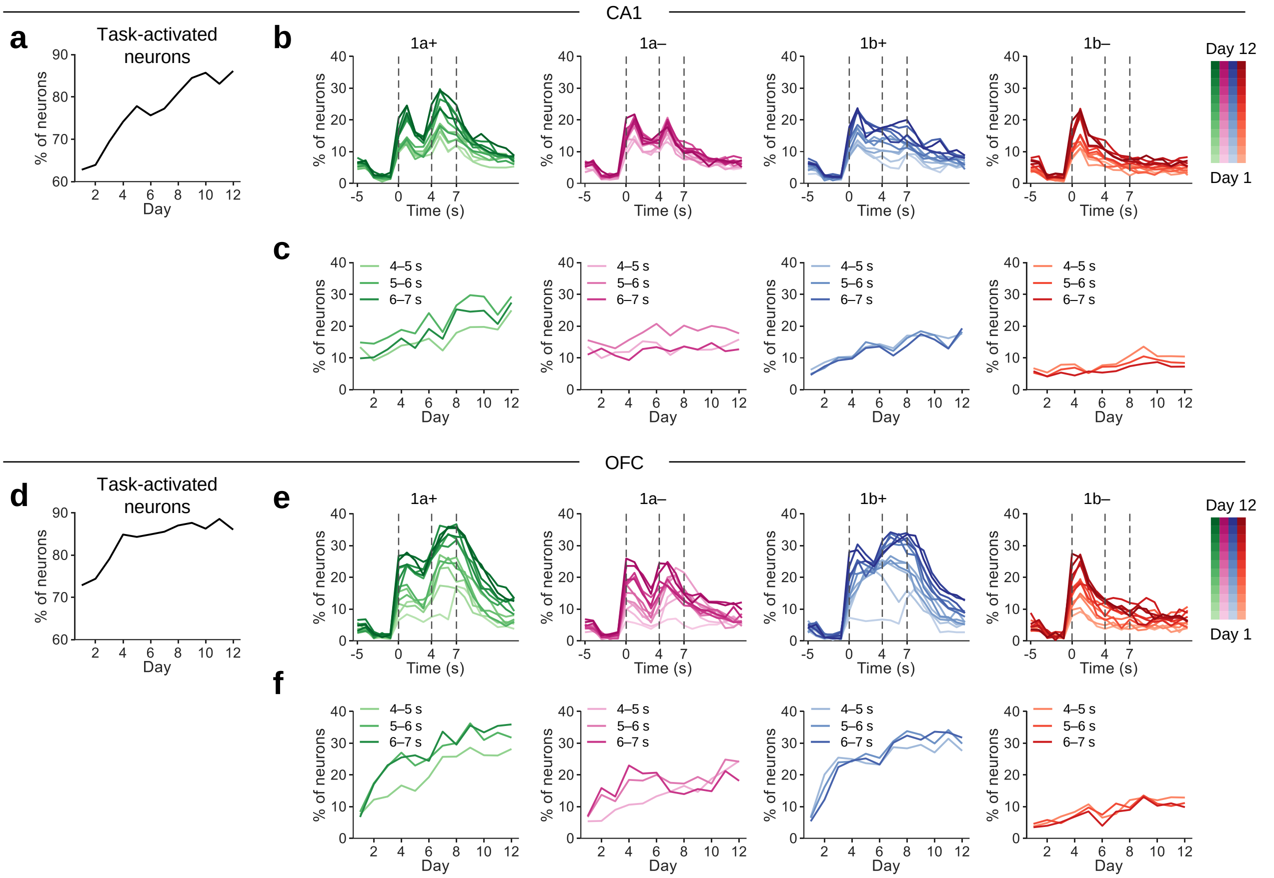

Supplementary Fig. 3 | Task-related neural activations in CA1 and OFC during the learning of Problem 1. **a** Fraction of neurons exhibiting activities significantly different from the baseline (2 s before the onset of first odors) at the period during 0–15 s (bin size: 1 s; **p* < 0.01, Wilcoxon rank sum test). **b** Fractions of neurons showing activation in each 'odor-outcome' sequence (bin size: 1 s; **p* < 0.01, Wilcoxon rank sum test). Light to dark colors indicate training days. **c**, Fractions of neurons showing activation in each of the four sequences at different phases of the splitting time window (4–7 s) throughout learning (bin size: 1 s; **p* < 0.01, Wilcoxon rank sum test). **d**-**f** OFC neurons with the same analyses as in **a**-**c**.

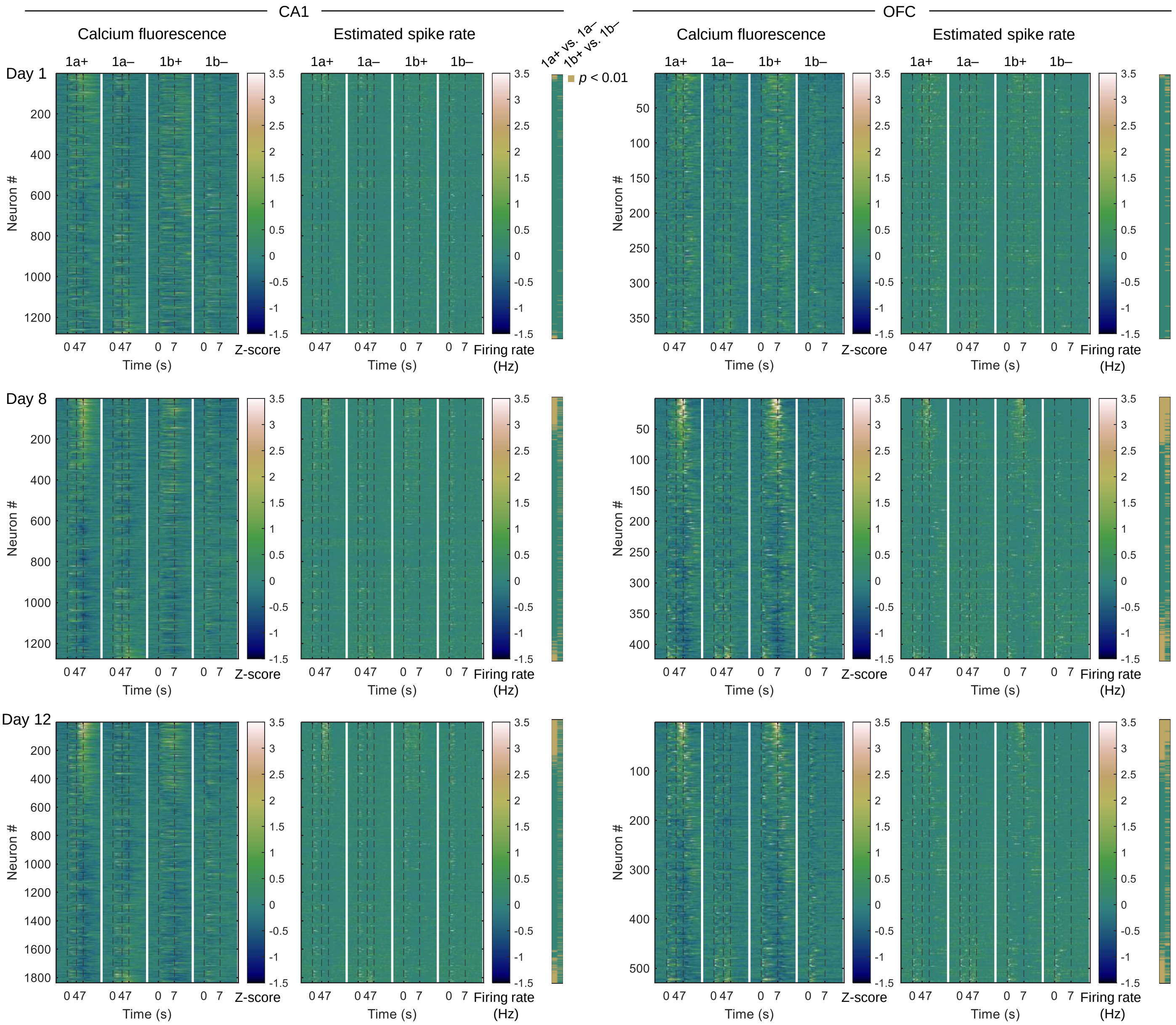

Supplementary Fig. 4 | Neural activities in CA1 and OFC during the learning of Problem 1. For each brain region, the two columns of heatmaps show the mean fluorescent signals in *z*-score and estimated spike rates, respectively. Each row represents one neuron. The four odor sequences were divided by white vertical lines. The neurons were ranked by their AUC (area under the ROC curve) that calculated their differential activities to 1a+ and 1a– during the second odor or delay period (4–7 s), using the spike rate data. The dashed lines indicate the time points of events (odors and outcomes) in the sequence. The right column shows the statistical significance of differential neural activities to each sequence pair (*$p < 0.01$, Wilcoxon rank sum test).

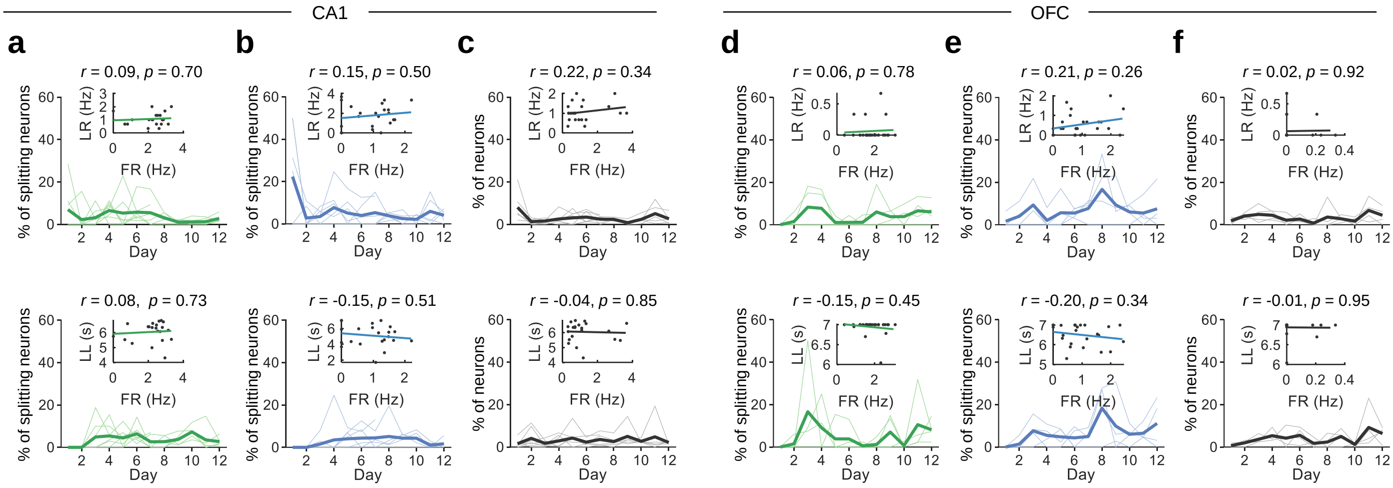

Supplementary Fig. 5 | Correlations of neural activities and licking behaviors. **a**-**c** Fractions of CA1 odor-splitting neurons (**a**), delay-splitting neurons (**b**), and all neurons (**c**) whose firing rates (FR) were significantly correlated with the lick rate (LR) and lick latency (LL) at 4–7 s, with the scatter plots of the same example neurons for each column inserted (*p < 0.01, Pearson's correlation). **d**-**f** OFC neurons with the same analysis as in **a**-**c**.

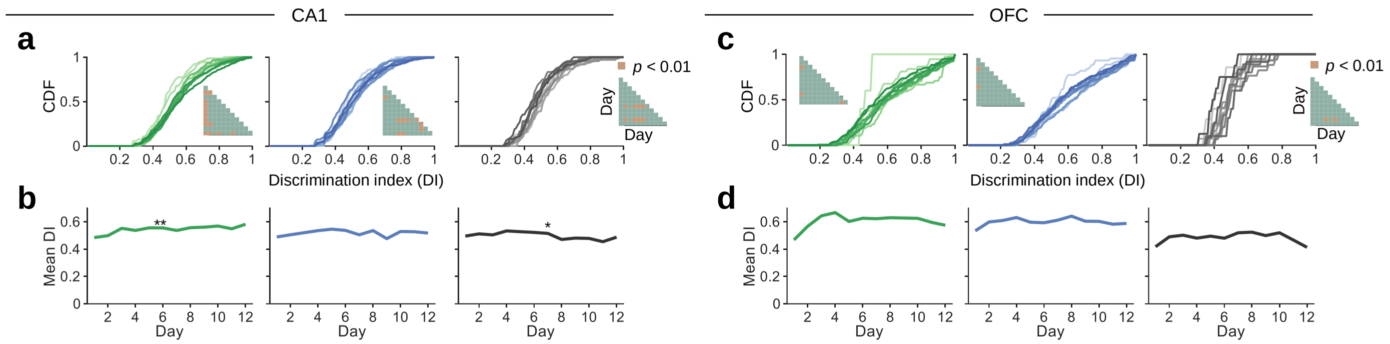

Supplementary Fig. 6 | Changes in the splitting strength of splitting neurons during learning of Problem 1. **a** The cumulative distribution function (CDF) of the discrimination index (DI) of CA1 splitting neurons at overlapping odor, delay, and reward periods. Inserted lower triangles are the pairwise comparison between the DI distributions of two training days using the Kolmogorov-Smirnov test. **b** The mean DI of splitting neurons in the CA1 during learning. The correlation between mean DIs and training days was tested using Spearman's rank correlation. *$p < 0.05$, **$p < 0.01$. **c**-**d** Splitting neurons in the OFC with the same analyses as in **a** and **b**, respectively.

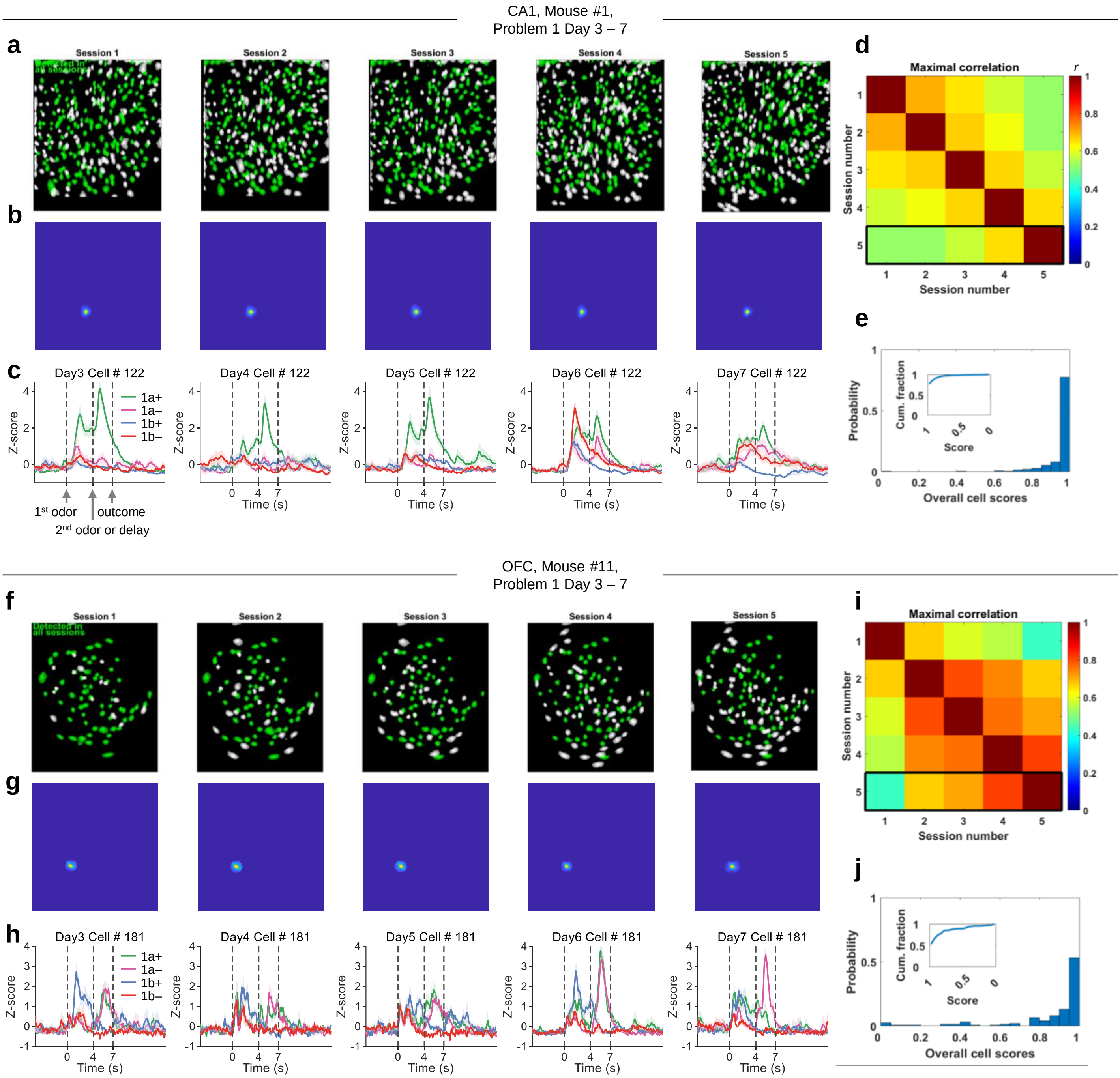

Supplementary Fig. 7 | Cell registration during mice learning Problem 1. a Spatial footprints of detected neurons in 5 imaging days of a representative mouse from CA1 group, with cells (labeled in green) that existed throughout 5 sessions. **b**-**c** The spatial configuration (**b**) and task-aligned activities (**c**) of an example aligned neuron. **d** Maximal cross-correlations between sessions by translations and rotations of the spatial footprints of each session. Black rectangle indicates the reference session. **e** The distribution of register scores, computed by the false positives, false negatives and non-exclusive cell registrations estimated by the model across all session pairs to assess the registration accuracy. **f**-**j** Alignment of OFC neurons during mice learning Problem 1 using the same format as in **a**-**e.**

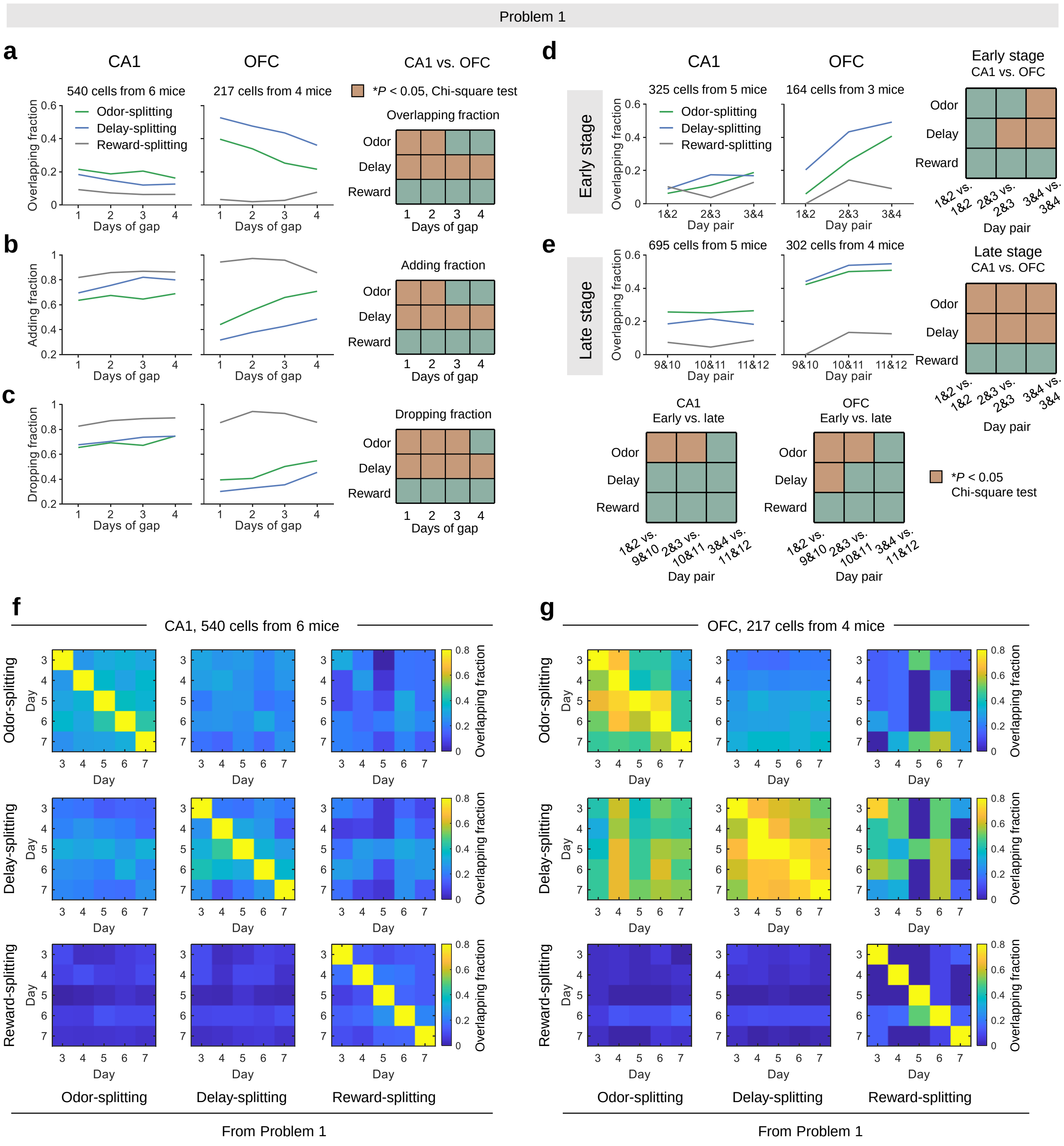

Supplementary Fig. 8 | Longitudinal analyses during mice learning Problem 1. a-c Overlapping (**a**), adding (**b**), and dropping (**c**) fractions of splitting neurons in CA1 and OFC across days as a function of days of gap averaged from Day 3 to Day 7 in Problem 1. The differences between the two brain regions were examined by Chi-square test (*$p < 0.05$). **d-e** The overlapping fractions of splitting neurons in the CA1 and OFC during the early stage (**d**, Day 1 to Day 4) and the late stage (**e**, Day 9 to Day 12) of Problem 1 training. Chi-square tests were performed to examine the differences between learning stages and between brain regions (*$p < 0.05$). **f-g** Fractions of CA1 (**f**) and OFC (**g**) neurons that were splitting neurons in each event and a given day (*x*-axes) also showed splitting activities in other events (*y*-axes) across days from Day 3–7 in Problem 1.

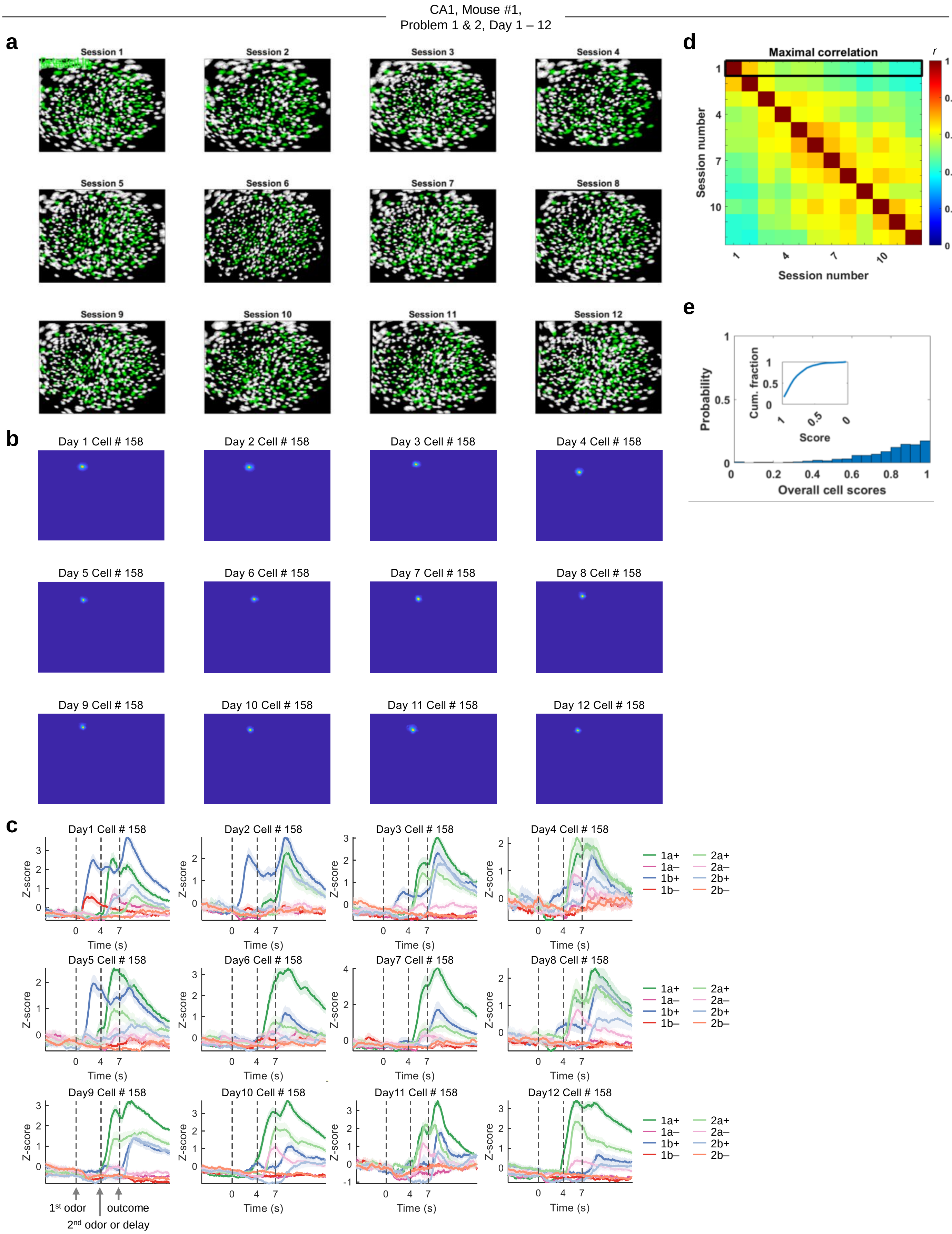

Supplementary Fig. 9 | Registration of CA1 neurons during the phase of Problem 1 & 2. a-**e**, Alignment of CA1 neurons during Problem 1 & 2 (Day 1 to Day 12) using the same format as in Supplementary Fig. **7a**-**e**.

OFC, Mouse #11,
Problem 1 & 2, Day 1 – 12

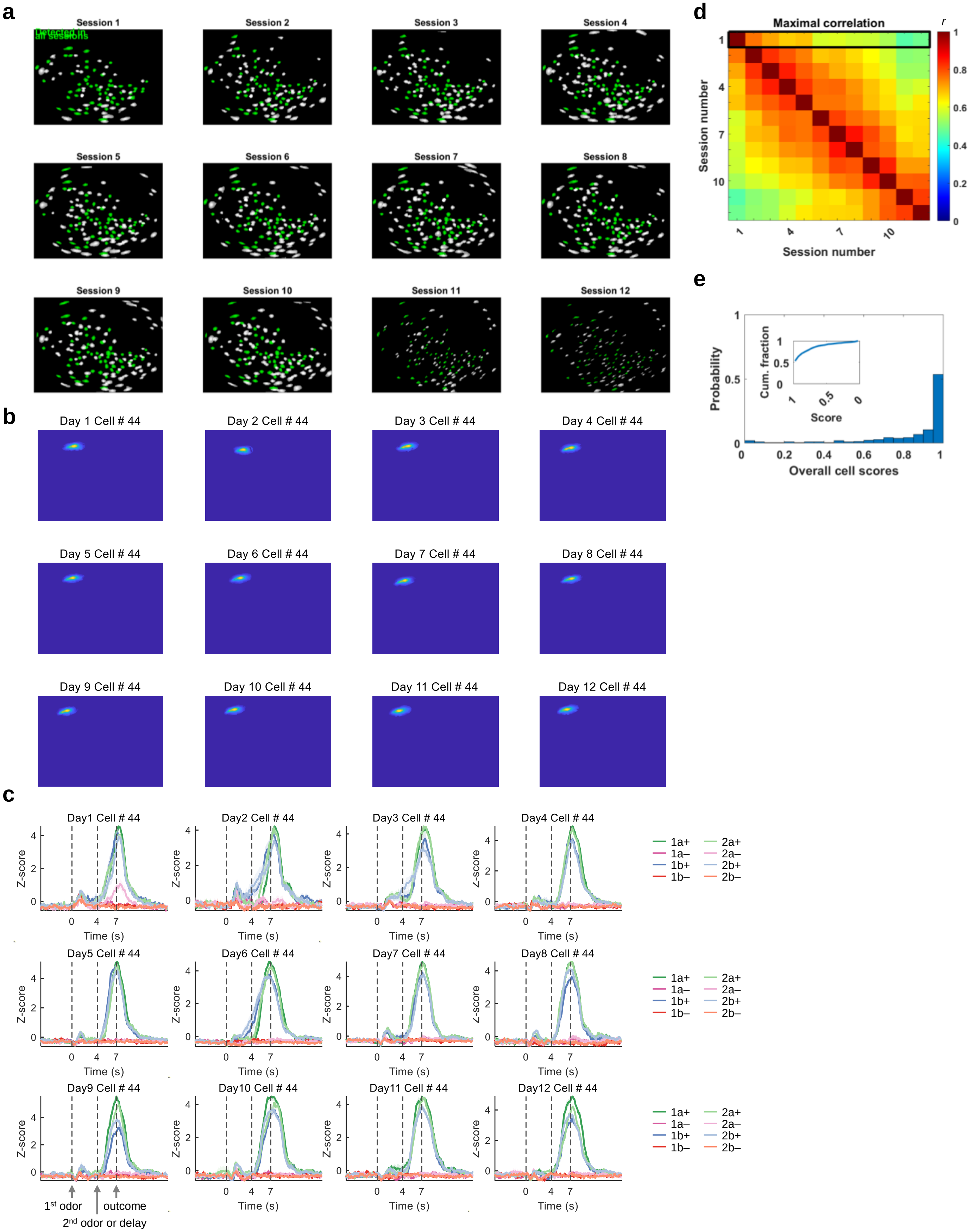

Supplementary Fig. 10 | Registration of OFC neurons during the phase of Problem 1 & 2. a-e, Alignments of OFC neurons during Problem 1 & 2 (Day 1 to Day 12) using the same format as in Supplementary Fig. **7a-e**.

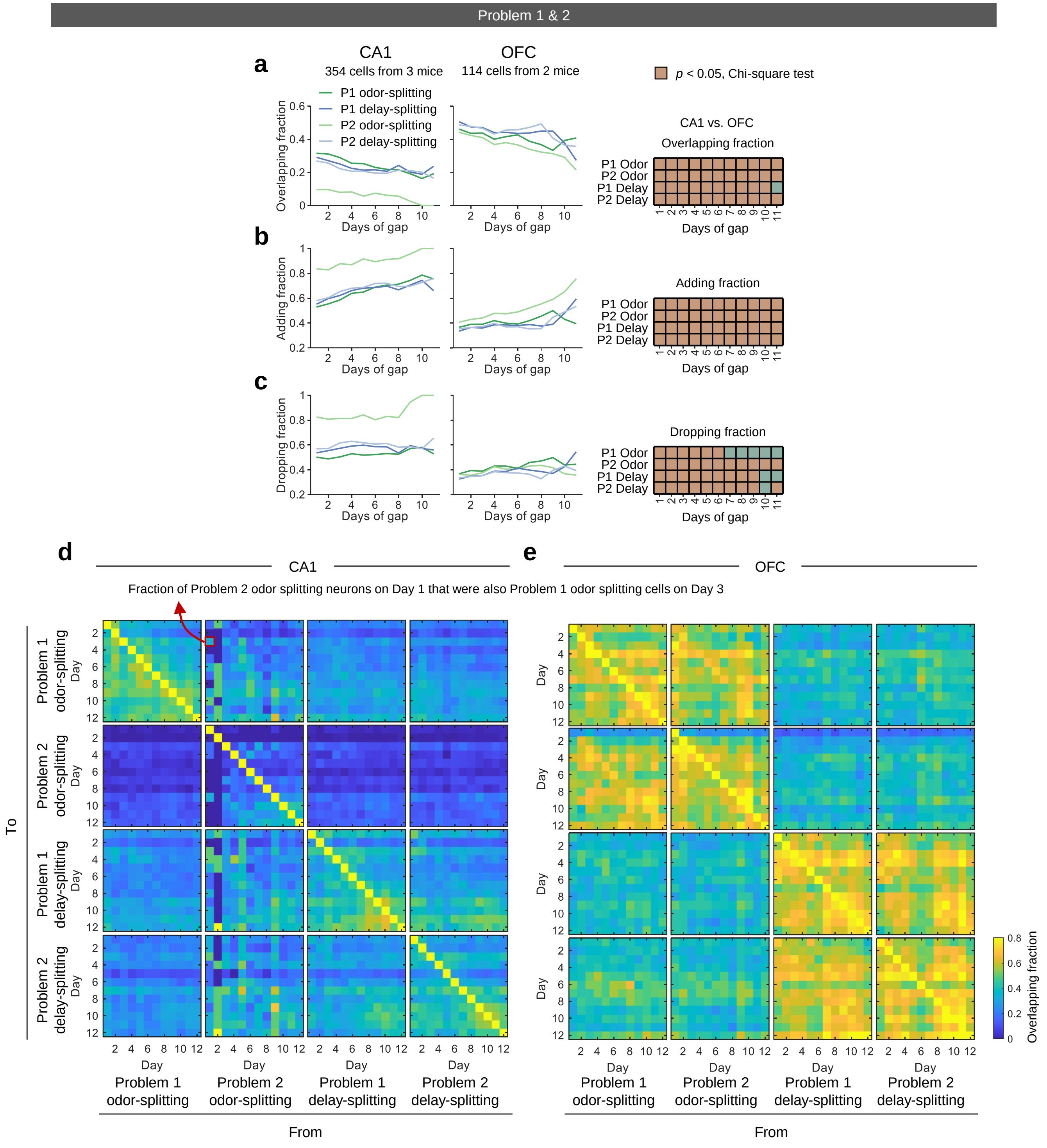

Supplementary Fig. 11 | Longitudinal analyses during learning of Problem 1 & 2. Splitting neurons across 12 days of Problem 1 & 2 with the same analyses as in Supplementary Fig. 8. **a**-**c** Overlapping (**a**), adding (**b**), and dropping (**c**) fractions of splitting neurons in CA1 and OFC across days as a function of days of gap averaged across 12 days in Problem 1 & 2. The differences between the two brain regions were examined by Chi-square test (**p* < 0.05). **d**-**e** Fractions of CA1 (**d**) and OFC (**e**) neurons that were splitting neurons in each event and a given day (*x*-axes) also showed splitting activities in other events (*y*-axes) across days from Problem 1 & 2.

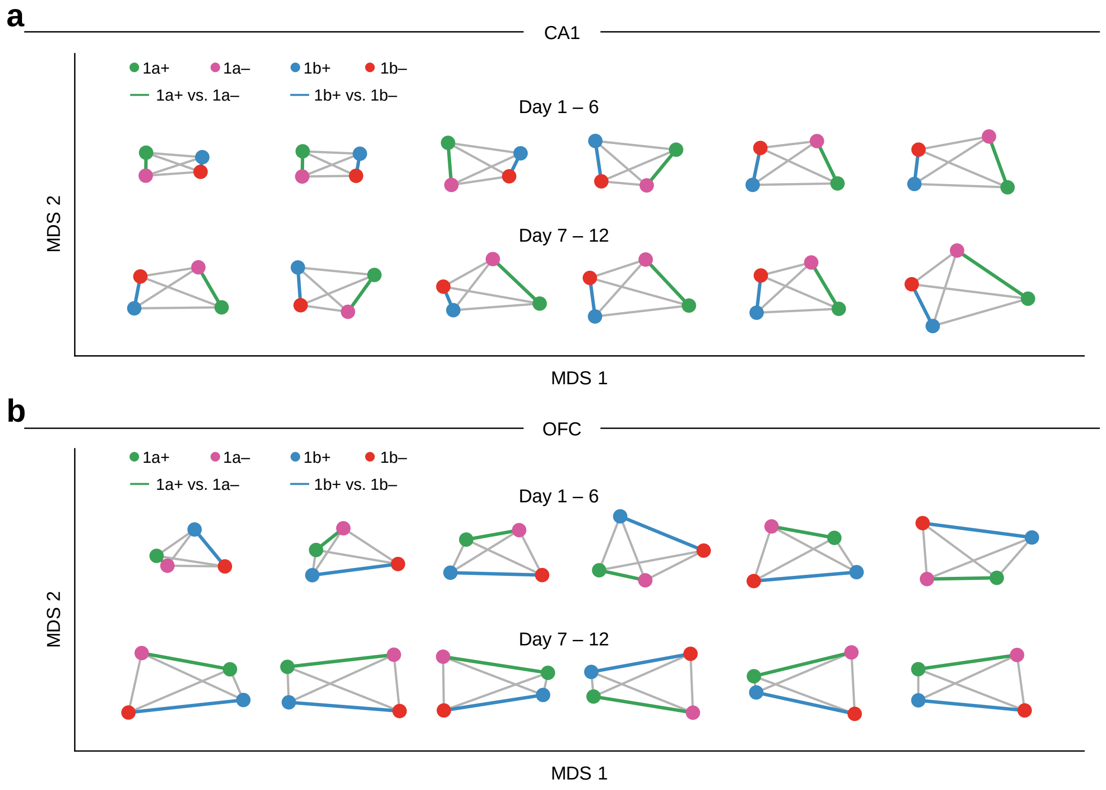

Supplementary Fig. 12 | Geometric visualization of splitting signals in Problem 1 through multidimensional scaling (MDS). **a**-**b** Projections of high-dimensional neural activities in CA1 (**a**) and OFC (**b**) related to four sequences (1a+, 1a–, 1b+, 1b–) during overlapping epochs (4–7 s) onto a two-dimensional neural activity space using the MDS.

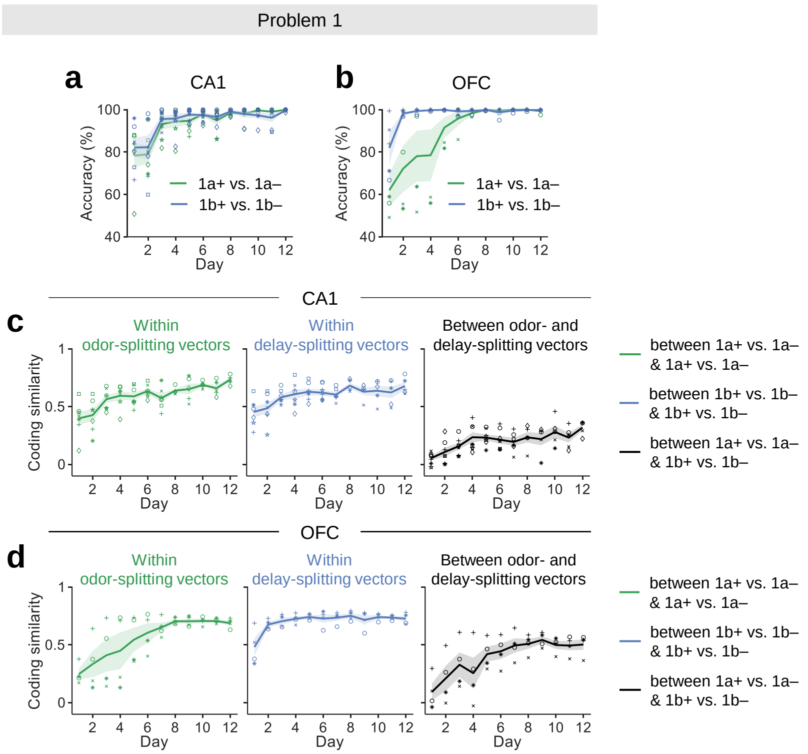

Supplementary Fig. 13 | Neural ensemble analyses on individual sessions produced results consistent with pseudo-ensemble analyses on Problem 1, related to Fig.4. a-**b** SVM decoding for paired sequences trained on neural activity during 4–7s with all neurons recorded in individual sessions of Problem 1 from CA1 (**a**) and OFC (**b**). The accuracy was averaged over 500 repeats on each session, with green and blue indicating odor and delay sequence pairs, respectively. **c**-**d** Coding similarity of splitting signals within odor-splitting, within delay-splitting, and between odor- and delay-splitting vectors in CA1 (**c**) and OFC (**d**), averaged across 300 repeats. For **a**-**d**, each marker represents a session, and the marker types indicate animal IDs. Lines and shaded areas indicate the mean decoding accuracy and mean ± SEM, respectively.

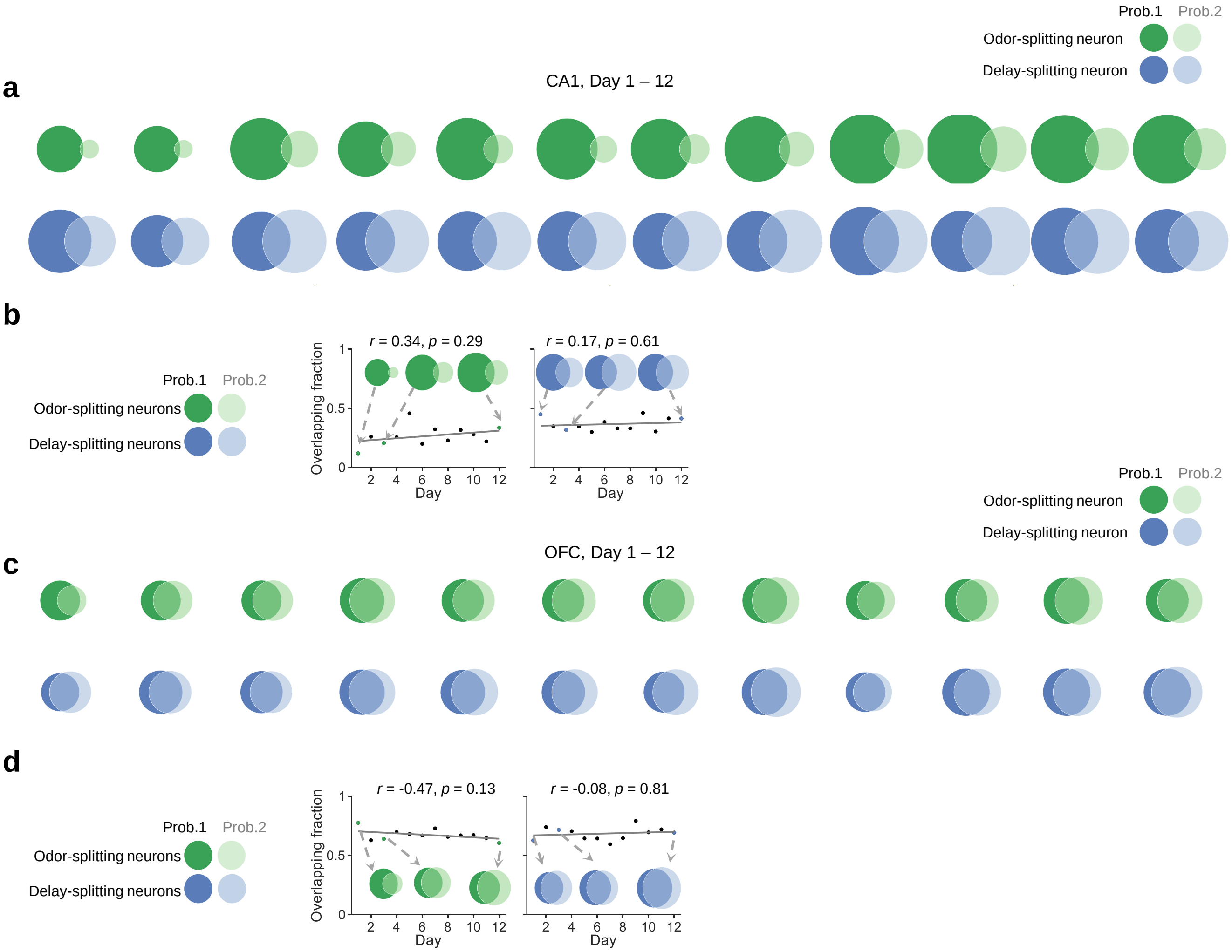

Supplementary Fig. 14 | Overlapping fractions of splitting neurons in CA1 and OFC during mice learning Problem 1 & 2. **a**-**b** Venn diagrams of odor- and delay-splitting neurons in Problem 1 and Problem 2 in CA1 (**a**) and OFC (**b**). **c**-**d**, Overlapping fractions of splitting neurons in CA1 (**c**) and OFC (**d**) during learning, with the denominator being the splitting neurons in Problem 2. Colored dots show example sessions with Venn diagrams. Spearman's rank correlation tested correlations between overlapping fractions and training days.

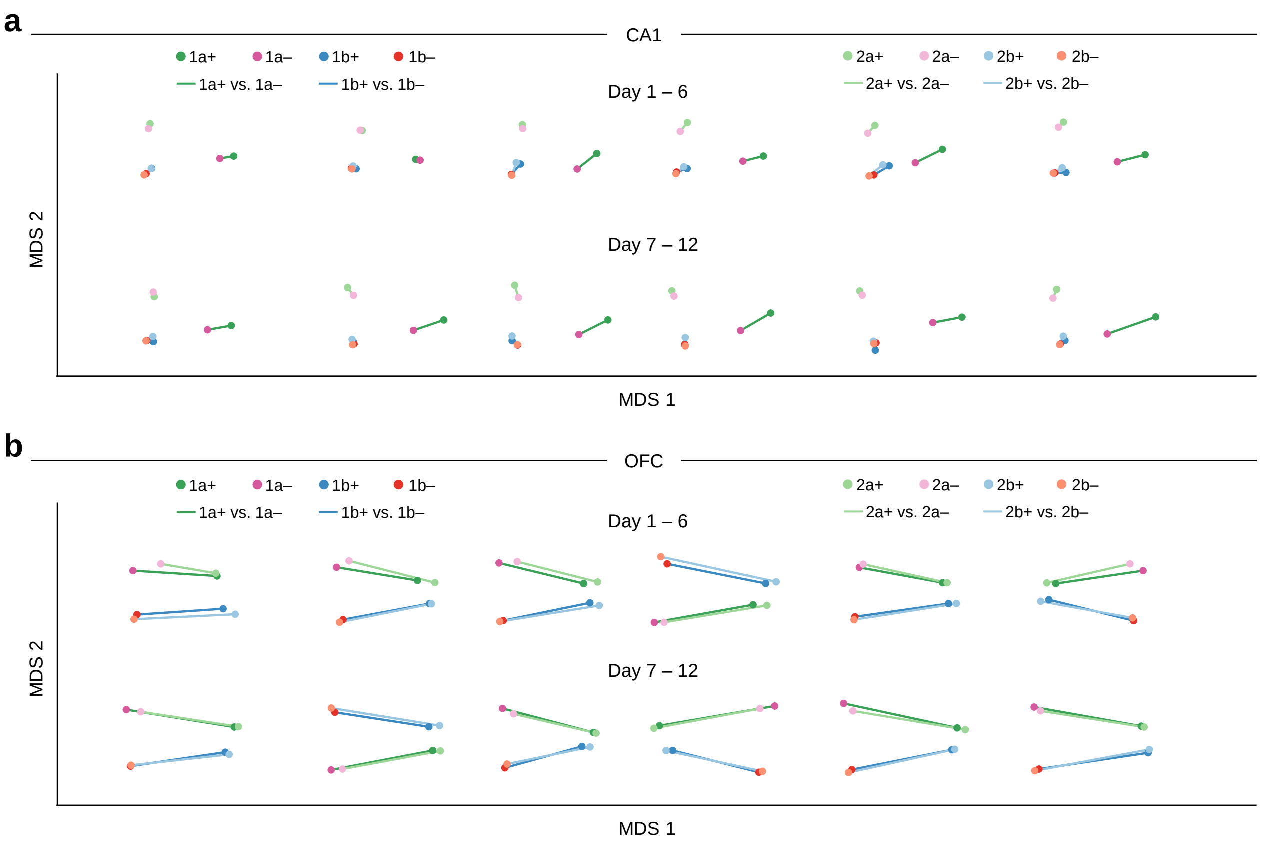

Supplementary Fig. 15 | Geometric visualization of splitting signals during mice learning Problem 1 & 2. **a**-**b** Projections of high-dimensional neural activities in CA1 (**a**) and OFC (**b**) related to 8 odor sequences (1a+, 1a–, 1b+, 1b–, 2a+, 2a–, 2b+, 2b–) during overlapping epochs (4–7 s) onto two-dimensional space using the MDS.

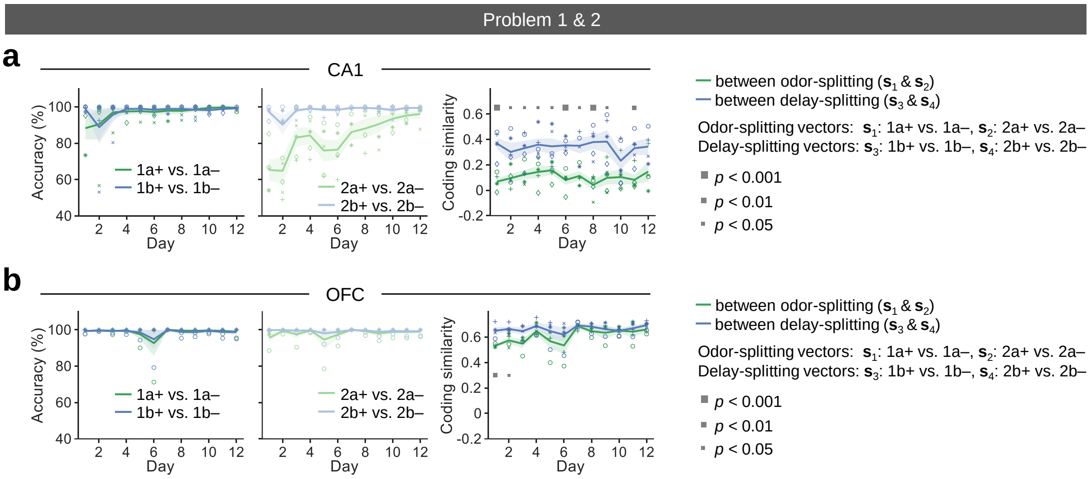

Supplementary Fig. 16 | Neural ensemble analyses on individual sessions produced results consistent with pseudo-ensemble analyses on Problem 1 & 2, related to Fig. 6. a-**b** SVM decoding for paired sequences trained on neural activity during 4–7s with all neurons recorded in individual sessions and the coding similarities between two odor and two delay splitting vectors from CA1 (**a**) and OFC (**b**) during the training of Problem 1 & 2, with the same format as in Supplementary Fig. 12.

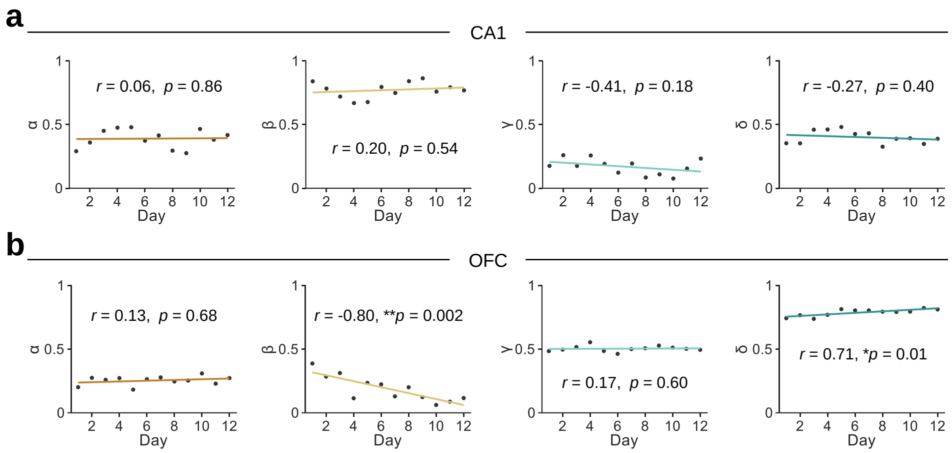

Supplementary Fig. 17 | Model fitting reveals the contributions of four task variables to CA1 and OFC splitting signal generalization during two-problem training. **a**-**b** Estimated weights of past cue (α), current cue (β), sequence structure (γ), and expected outcome (δ) for CA1 (**a**) and OFC (**b**) during training of two problems. Dots are the averages of 100 repeats of estimated parameters on each day. Spearman's rank correlation tested correlations between the weights and training days.

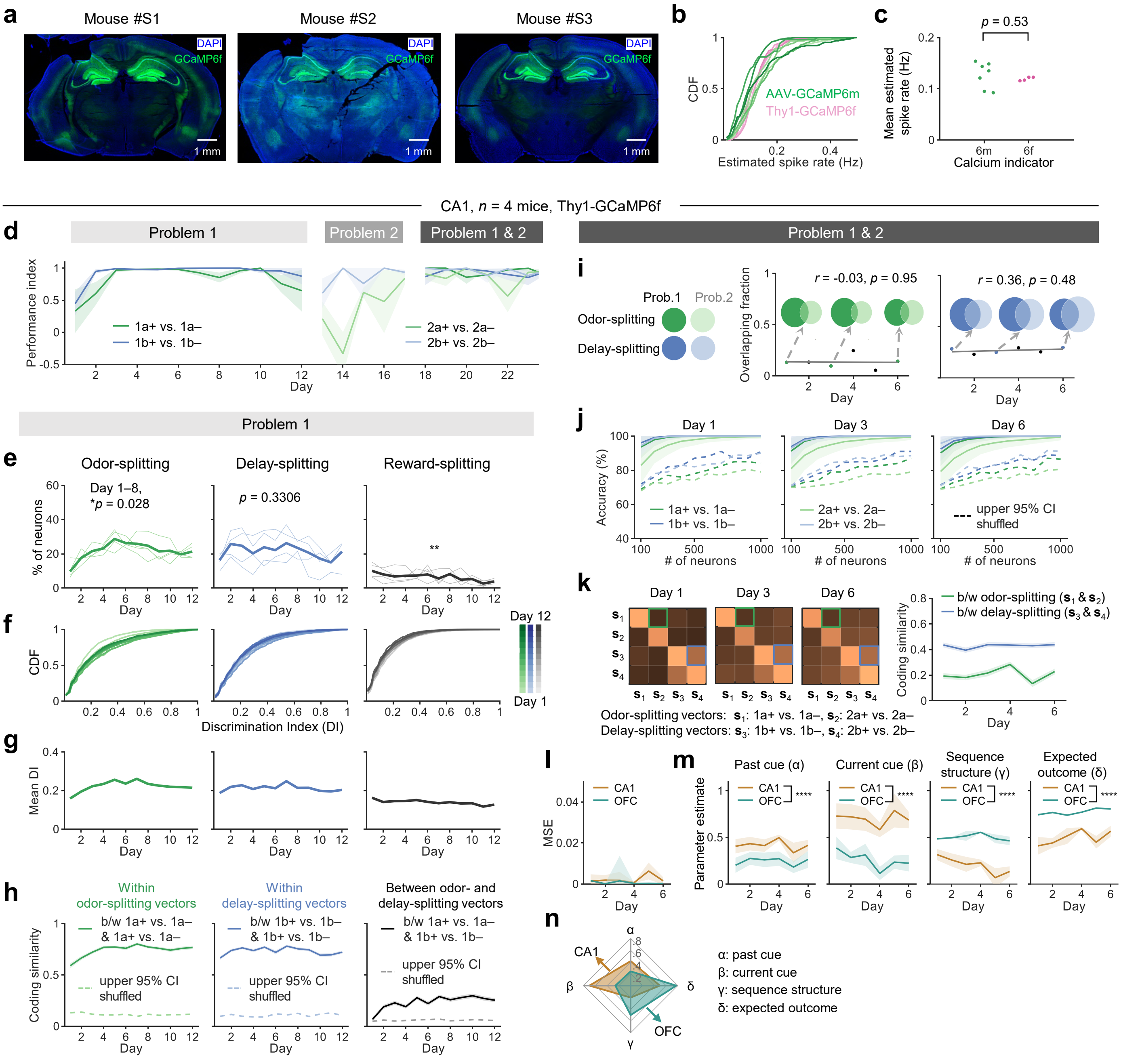

Supplementary Fig. 18 | Findings from CA1 imaging with AAV expressing GCaMP6m were replicated by imaging with Thy1-GCaMP6f mice. a GRIN lens placement in the CA1 of Thy1-GCaMP6f mice. **b-c** Estimated spike rates of imaged CA1 neurons between two groups of mice with different calcium indicators, GCaMP6m (wild-type mice with AAV delivery) and GCaMP6f (Thy1-CaMP6f mice), showed no significant differences in terms of estimated spike rate distributions and the mean estimated spike rates (Wilcoxon rank sum test). **d** The learning curve of sequence discrimination, analyzed in the same way as in **Fig. 1d** upper panel. Note that before the current training, these mice had also been briefly trained with odor-reward associations in a prior pilot experiment using different odor cues, which might explain their faster learning than the mice injected with AAV-GCaMP6m mice. **e** Odor-, delay- and reward-splitting neuron fractions during learning of Problem 1 with the same analysis as in **Fig. 3c**. **f** The cumulative distribution function (CDF) of the discrimination index (DI) of CA1 neurons at overlapping odor, delay, and reward periods. **g** The mean DI of all CA1 neurons during learning. **h** Coding similarity of splitting signals within odor-splitting, within delay-splitting, and between odor- and delay-splitting vectors, averaged across 300 repeats, as in **Fig. 4c**. **i** Overlapping fraction of splitting neurons in CA1 during learning, with the same format as **Fig. 6a**. **j** SVM decoding for paired sequences trained on the neural activity during 4–7 s with randomly sampled neurons in CA1, as in **Fig. 6b**. **k** Coding similarity of four splitting signals in CA1 during learning. **l** Recovered parameters to fit the model in the CA1 and OFC of Thy1-GCaMP6f mice. Shaded areas indicate SD (n = 100 repeats). The significance of differences for each parameter was examined by two-way ANOVA (‘brain region’ and ‘day’, ****$p < 0.0001$). **m** MSE between the predicted and actual coding similarity in CA1 and OFC. Shaded areas indicate SD (n = 100 repeats). **n** Radar plot of parameter estimates averaged across 6 days in CA1 and OFC. **l**, **m, n** Parallel analyses to the **Fig. 7c**, **d**, **e**.

Supplementary Table 1. The number of neurons recorded during the experiment.

	CA1, AAV-hSyn-GCaMP6m								OFC, Thy1-GCaMP6f					CA1, Thy1-GCaMP6f				
Mouse #	1	2	3	4	5	6	7	Total	8	9	10	11	Total	S1	S2	S3	S4	Total
Day 1	225	90	214	159	53	407	132	1280	21	139	109	103	372	490	349	274	294	1407
Day 2	255	58	188	184	44	333	128	1190	35	126	81	86	328	455	341	279	250	1325
Day 3	368	116	224	222	65	370	167	1532	40	117	95	82	334	494	368	301	292	1455
Day 4	367	105	176	210	77	373	200	1508	32	139	132	93	396	438	337	357	270	1402
Day 5	368	87	199	221	94	372	210	1551	46	136	116	103	401	446	373	331	300	1450
Day 6	422	160	221	307	128	358	272	1868	38	147	128	117	430	435	362	310	285	1392
Day 7	437	161	n/a	300	123	350	263	1634	35	136	128	129	428	402	354	315	251	1322
Day 8	462	158	n/a	256	129	n/a	270	1275	46	138	119	120	423	491	396	306	277	1470
Day 9	461	204	n/a	360	176	n/a	299	1500	36	148	118	142	444	424	410	287	292	1413
Day 10	510	252	n/a	309	160	n/a	350	1581	51	136	135	136	458	416	390	278	265	1349
Day 11	491	257	n/a	305	186	n/a	358	1597	52	125	156	138	471	418	375	295	293	1381
Day 12	564	324	n/a	359	221	n/a	368	1836	66	162	159	142	529	476	403	249	316	1444
Day 13	629	341	n/a	346	255	n/a	419	1990	44	149	163	152	508	420	337	314	293	1364
Day 14	662	300	n/a	256	223	n/a	414	1855	51	150	157	130	488	446	389	305	276	1416
Day 15	577	311	n/a	325	232	n/a	376	1821	56	148	152	131	487	428	354	281	275	1338
Day 16	484	283	n/a	260	188	n/a	0	1215	60	153	161	134	508	390	378	344	286	1398
Day 17	523	262	n/a	0	231	n/a	411	1427	50	166	164	84	464	428	377	265	319	1389
Day 18	539	317	n/a	285	289	n/a	432	1862	45	126	125	94	390	455	401	300	309	1465
Day 19	492	234	n/a	305	243	n/a	394	1668	51	109	134	127	421	427	448	312	311	1498
Day 20	574	323	n/a	260	254	n/a	308	1719	41	135	124	132	432	429	435	300	362	1526
Day 21	471	290	n/a	277	292	n/a	274	1604	44	128	143	141	456	438	408	246	339	1431
Day 22	591	300	n/a	267	284	n/a	297	1739	39	166	163	132	500	429	411	273	298	1411
Day 23	575	327	n/a	287	299	n/a	241	1729	42	130	153	136	461	507	369	300	311	1487
Day 24	599	343	n/a	0	292	n/a	291	1525	0	133	145	144	422	n/a	n/a	n/a	n/a	n/a
Day 25	572	352	n/a	243	270	n/a	292	1729	52	143	142	137	474	n/a	n/a	n/a	n/a	n/a
Day 26	602	293	n/a	213	260	n/a	286	1654	44	0	151	139	334	n/a	n/a	n/a	n/a	n/a
Day 27	568	373	n/a	143	264	n/a	307	1655	47	139	176	146	508	n/a	n/a	n/a	n/a	n/a
Day 28	574	377	n/a	137	291	n/a	313	1692	51	140	168	127	486	n/a	n/a	n/a	n/a	n/a
Day 29	590	379	n/a	0	293	n/a	271	1533	41	154	157	139	491	n/a	n/a	n/a	n/a	n/a
